# Supplementary material for: Deep transfer learning-based bird species classification using mel spectrogram images
Source: PLoS One. 2024 Aug 12;19(8):e0305708. doi: 10.1371/journal.pone.0305708 (PMC11318847; doi:10.1371/journal.pone.0305708)
Supplement: Attachment — Submitted filename: response-to-reviewers.R1.pdf [file pone.0305708.s001.pdf]

Note: The blue-colored text inside the brown-colored box is the revised text. ~~Red-colored Strikethrough text~~ means those texts have been removed in this revision. All the removed texts have not been shown.

Reviewer #1:

Comment 1: In abstract, the authors mentioned background in present form. Is that current challenges of the technique of previous challenge?

Authors response: The background mentioned has been converted to the past form, and the current solutions performed in this research have been mentioned in the present form.

Authors action: We have revised the abstract. The changes are visible in blue text excerpts from the revised manuscript (location: abstract).

> The classification of bird species is of significant importance in the field of ornithology, as it plays an important role in assessing and monitoring environmental dynamics, including habitat modifications, migratory behaviors, levels of pollution, and disease occurrences. Traditional methods of bird classification, such as visual identification, were time-intensive and required a high level of expertise. However, audio-based bird species classification is a promising approach that can be used to automate bird species identification. This study aims to establish an audio-based bird species classification system for 264 Eastern African bird species employing modified deep transfer learning. In particular, the pre-trained EfficientNet technique was utilized for the investigation. The study adapts the fine-tune model to learn the pertinent patterns from mel spectrogram images specific to this bird species classification task. The fine-tuned EfficientNet model combined with a type of Recurrent Neural Networks (RNNs) namely Gated Recurrent Unit (GRU) and Long short-term memory (LSTM). RNNs are employed to capture the temporal dependencies in audio signals, thereby enhancing bird species classification accuracy. The dataset utilized in this work contains nearly 17,000 bird sound recordings across a diverse range of species. The experiment was conducted with several combinations of EfficientNet and RNNs, and EfficientNet-B7 with GRU surpasses other experimental models with an accuracy of 84.03% and a macro-average precision score of 0.8342.

Comment 2: "In this study, our aim is to develop an audio-based bird species" statement is not upto the mark.

Authors response: The statement has been standardized as opposed to the previous one.

Authors action: We have revised the statement, and, in the following, the changes are excerpted from the revised manuscript (location: abstract).

> identification. This study aims to establish an audio-based bird species classification system for 264 Eastern African bird species employing modified deep transfer learning.

Comment 3: Authors must not use words like "we", "me", "I", "if" etc.

Authors response: "we", "me", and "I" are replaced with passive forms. We also took necessary steps where the word "if" was introduced.

Authors action: We have revised the manuscript to implement this comment. In the following, we have excerpted (a sample of the changes for this comment) from the revised manuscript.

> This study aims to develop an accurate and efficient audio-based bird species classification system by using mel spectrogram images and deep learning techniques. The study is divided into several steps. First, ~~we collect~~ the BirdCLEF2023 dataset is collected, which focuses on identifying bird calls in soundscapes, from Kaggle [35]. Subsequently, ~~we perform~~ pre-processing steps are performed, which involve converting audio data into mel spectrogram images. ~~We then perform a dataset~~ In addition our dataset is split into training, testing, and validation sets and ~~apply~~ applied random upsampling on the training set to minority classes in order to solve class imbalance

---

Comment 4: "In particular, we explore using EfficientNet, a pre-trained convolutional neural network (CNN) originally trained using mel spectrogram images derived from bird sound recordings." structure is not upto the mark.

Authors response: We apologize for any inconvenience caused. To enable the statement to properly convey its content, the sentence's structure was adjusted while maintaining a simple form.

Authors action: We have revised the manuscript to implement this comment. The change is visible in the following sentence excerpted from the revised manuscript (location: abstract).

> In particular, the pre-trained EfficientNet technique was utilized for the investigation.

Comment 5: somewhere they mentioned 264 bird species and somewhere 17,000 bird sounds recording. Statement is not clear.

Authors response: "264 bird species" denotes there are 264 types of birds.

To our knowledge, we did not use the phrase '17,000 bird sounds recordings', but we used 'nearly 17,000 bird sounds recordings'. Referring to Table I, the exact bird sound recordings are 16753. Therefore, both are correct according to our perspective. In the following, we have excerpted from the revised manuscript a sample of the concept in Table 1.

Table 1. Basic statistics of dataset

Items	Value
Number of bird sounds / audio recordings	16753
Number of bird species	264
Number of sampling rate of audios	32000 Hz

Authors action: We did not revise the manuscript for this comment.

Comment 6: "surpasses competing models with an accuracy of 84.03\% and a macro-average precision score of 0.8342." typo error in this statement.

Authors response: We compare the model performance in comparison with the model that we experimented with. We did not find any existing publications on the datasets we worked with. Therefore, we cannot compare it to other competing models. Therefore, the portion we have written as "surpassing competing models", we updated with "surpassing experimental models".

Authors action: To address this comment we revised the manuscript as follows (location: abstract).

of species. ~~We experimented~~ The experiment was conducted with several combinations of EfficientNet and RNNs, and EfficientNet-B7 with GRU surpasses ~~competing~~ other experimental models with an accuracy of 84.03% and a macro-average precision score of 0.8342.

Comment 7: Figures are missing, only captions are there.

Authors response: We must follow the template according to the journal guidelines. According to the journal, in the text only the caption should be located. The Figure with the caption is given at the end of the file.

Authors action: We did not revise the manuscript for this comment.

Comment 8: It seems the authors were hasty while writing the manuscript. They are suggested to read the whole manuscript carefully before submission.

Authors response: We apologize for the inconvenience caused. The whole manuscript has been carefully read before submitting the revised version.

Authors action: We carefully read the manuscript and amended it where necessary.

Reviewer #2:

Comment 1: The parameters used to obtain the Mel-spectrogram should be given.

Authors response: We have provided parameters for the mel spectrogram images in Table 2.

Authors action: In the following, we have excerpted from the revised manuscript a sample of the parameters for obtaining mel spectrogram images in Table 2 shown below.

Table 2. Table of Parameters and Values

Parameter	Value
Samples Between Consecutive Windows	2048
Number of Mel Frequency Bands	128
Minimum frequency of the Mel Filter Bank (Hz)	500
Maximum frequency of the Mel Filter Bank (Hz)	12500
Centered	False

Comment 2: Why Mel-spectrogram features are preferred should be explained in more detail.

Authors response: Mel spectrogram features are favored in audio signal processing because of their ability to closely mimic the human auditory system's response to sound, employing a nonlinear frequency scale known as the mel scale. This characteristic enables mel-spectrograms to better capture the perceptual aspects of audio, particularly in tasks involving human speech and music. Additionally, mel-spectrograms offer a more compact representation of spectral content compared to traditional spectrograms, as they group frequencies into logarithmically spaced mel frequency bands, reducing dimensionality and improving computational efficiency for machine learning algorithms.

Authors action: We revised the manuscript for this comment. The following is the excerpted part of the revised manuscript that is related to the response of this comment.

> mel-spectrograms that potentially have a significant impact on the analysis. Mel spectrogram images offer a valuable approach for classifying bird species through audio data due to their ability to represent frequency patterns effectively. By breaking down audio signals into distinct frequency components over time, mel-spectrograms serve as a powerful feature extraction technique [13]. Mel-spectrograms also provide a visually interpretable representation, aiding in both model validation and human analysis of acoustic characteristics. Furthermore, their dimensionality reduction compared to raw waveforms facilitates computational efficiency. This approach has demonstrated success in various audio classification tasks, making it a reliable choice for bird species identification. Authors in [14] shows that mel spectrogram images perform better as

---

Comment 3: The results need to be compared across literature.

Authors response: We discovered no published work on the dataset we worked with. That is why we were unable to demonstrate comparisons across different literatures.

Authors action: We did not revise the manuscript for this comment.

Comment 4: Perform k-fold cross-validation

Authors response: Our decision stems from the substantial computational demands associated with k-fold cross-validation, particularly in the context of our large dataset. Running multiple folds would lead to impractical training times, making it challenging to meet the study's objectives within reasonable timeframes.

To compensate, we adopted a stratified train-valid-test split, ensuring that both the training and validation sets maintain a representative distribution of each class. This approach not only addresses computational constraints but also upholds the generalizability of our model evaluation. We closely monitored the model's performance on the validation set during training to ensure effective prevention of overfitting. To establish the reliability of our model, we set a baseline performance using the test set. Our model's strong performance on this holdout test set underscores its ability to generalize effectively to unseen data.

Authors action: We did not revise the manuscript for this comment. The reason is already explained in the above '**Authors response**' part.
